# Early Diagnosis of Amyloidosis and Cardiac Involvement through Carpal Tunnel Surgery and Predictive Factors

**DOI:** 10.3390/jcm13154328

**Published:** 2024-07-24

**Authors:** María del Carmen Navarro-Saez, Carlos Feijoo-Massó, Alex Berenguer Sánchez, Tamara Parra Parente, Laura Guillamon Toran, Francesc Marcano-Fernández, Jaume Camara-Cabrera, Zully del Carmen Bravo Ferrer, Ricard Comet Monte, Xavier Calvet Calvo

**Affiliations:** 1 Acute Geriatric Unit and Infectious Diseases Department, Parc Taulí Hospital Universitari, Institut d’Investigació i Innovació Parc Taulí (I3PT), 08208 Sabadell, Spain; mnavarros@tauli.cat; 2 Internal Medicine Department, Parc Taulí Hospital Universitari, Institut d’Investigació i Innovació Parc Taulí (I3PT), 08208 Sabadell, Spain; rcomet@tauli.cat; 3 Hand Surgery Unit, Orthopaedic Surgery Department, Parc Taulí Hospital Universitari, Institut d’Investigació i Innovació Parc Taulí (I3PT), 08208 Sabadell, Spain; aberenguer@tauli.cat (A.B.S.); famarcano@tauli.cat (F.M.-F.); jcamara@tauli.cat (J.C.-C.); 4 Pathology Department, Parc Taulí Hospital Universitari, Institut d’Investigació i Innovació Parc Taulí (I3PT), 08208 Sabadell, Spain; tparra@tauli.cat; 5 Cardiology Department, Parc Taulí Hospital Universitari, Institut d’Investigació i Innovació Parc Taulí (I3PT), 08208 Sabadell, Spain; lguillamon@tauli.cat; 6 Nuclear Medicine Department, Parc Taulí Hospital Universitari, Institut d’Investigació i Innovació Parc Taulí (I3PT), 08208 Sabadell, Spain; zully.bravo@salutsantjoan.cat; 7 Gastroenterology Department, Parc Taulí Hospital Universitari, Institut d’Investigació i Innovació Parc Taulí (I3PT), 08208 Sabadell, Spain; xcalvet@tauli.cat

**Keywords:** amyloidosis, carpal tunnel syndrome, early diagnosis

## Abstract

**Background/Objectives**: To determine the prevalence of amyloidosis through the analysis of synovial tissue and transverse carpal ligament (TCL) in patients undergoing surgery for carpal tunnel syndrome (CTS), detect predictive factors for the presence of amyloid, and assess cardiac involvement degree. **Methods**: A prospective study with longitudinal cohort follow-up at a teaching hospital. Patients undergoing CTS surgery from 1 January 2019 to 31 May 2021 were included. Samples from synovial and TCL tissues were examined for amyloid presence. Multivariate analysis was used to detect predictive factors of the presence of amyloid. Patients with amyloid underwent echocardiography, laboratory analyses, and scintigraphy. **Results**: Two hundred and forty-six patients were included. The prevalence of amyloid was 11.4% in TCL and 12.6% in synovial tissues. Age (*p* = 0.035; OR 1.123), bilateral CTS symptoms (*p* = 0.022; OR 3.647), and trigger finger (*p* < 0.001; OR 3.537) were predictors of the presence of amyloid. Seventeen patients were diagnosed with transthyretin amyloidosis (ATTR) located in the carpus (no scintigraphic cardiac uptake or grade 0), one with light chain amyloidosis, eight with ATTR with cardiac involvement (grades 2–3), and five with ATTR in the carpus and scintigraphic uptake grade 1 (with normal echocardiogram and blood and urine tests). **Conclusions**: We detected amyloid in 12.6% of unselected consecutive patients who underwent CTS surgery. Biopsy in patients with CTS for amyloid detection, especially in elderly patients with bilateral symptoms and trigger finger, may be useful for the early diagnosis of amyloidosis, primarily due to transthyretin.

## 1. Introduction

Amyloidosis is a disease in which the abnormal folding of certain proteins affects the way they are deposited, causing structural and functional alterations in the involved organ. The type of precursor protein determines the amyloid subtype and also the organs affected [1,2].

The most common presentations are light chain amyloidosis, secondary amyloidosis, in which the precursor protein is protein A, amyloidosis secondary to hemodialysis, beta-2-microglobulin as the precursor protein, and amyloidosis produced by transthyretin deposition (ATTR), either via mutations or in its wild-type form.

Immunoglobulin light chains and transthyretin are two types of proteins that accumulate primarily in the myocardium, prompting complications such as heart failure or death [3].

Immunoglobulin light chains and transthyretin are the two types of proteins which primarily accumulate in the myocardium. In amyloidosis with cardiac involvement, cardiac symptoms usually appear in advanced stages of the disease, leading to later diagnosis and poor prognosis [4].

Cardiac amyloidosis presents with symptoms such as heart failure, arrhythmias, and conduction abnormalities. Echocardiography plays a vital role in differentiating various causes underlying the hypertrophic phenotype. Both standard and advanced echocardiographic techniques, including strain imaging, offer valuable insights for establishing an accurate diagnosis [5,6].

Classically, a definite diagnosis of cardiac amyloidosis required a mandatory histological confirmation. Currently, ATTR can also be diagnosed by excluding the presence of light chains in blood and urine added to confirmatory technetium-99m-labelled 3,3-diphosphono-1,2-propanodicarboxylic acid (99mTc-DPD) scintigraphy with myocardial uptake [7,8].

The treatment of amyloidosis varies according to its type, and its prognosis depends on the degree of cardiac involvement at the time of diagnosis. Prompt identification of cardiac amyloidosis is important because treatments have been shown to be effective in early stages of the disease [9,10,11]. In addition to myocardial deposition, a significant proportion of patients with cardiac amyloidosis show soft tissue deposition, which leads to manifestations such as lumbar canal stenosis, biceps tendon rupture, and bilateral carpal tunnel syndrome (CTS). Diagnosis is usually made when heart failure is already advanced; therefore, it is important to be alert to certain red flags that appear at earlier stages of the disease [12,13].

CTS is considered a “red flag” and precedes cardiac involvement by 5–9 years [12,13,14]. CTS is caused by the compression of the median nerve in the wrist. It is the most common peripheral focal neuropathy in the general population (prevalence of 1–5%). It may have various causes, including amyloidosis, but the most common is idiopathic [14]. Its treatment is usually based on surgical release of the median nerve by sectioning the structure that covers it, the transverse carpal ligament (TCL).

CTS is a common finding among patients with cardiac amyloidosis, especially if it is bilateral (38–48% of patients with wild-type ATTR, and 8–13% of patients with light chain-related amyloidosis) [15,16,17,18,19]. CTS is the initial manifestation in 20–25% of cases of cardiac amyloidosis [20,21]. Given that CTS precedes cardiac and systemic amyloidosis by several years, it can be used as an early marker to identify patients with cardiac involvement. Additionally, in patients with amyloidosis located in the carpus without cardiac involvement, the proportion that will develop cardiac amyloidosis in the future is unknown.

The main objective of this study was to determine the prevalence of amyloidosis through analysis of synovial tissue and TCL in patients undergoing carpal tunnel release surgery. Moreover, we aimed to identify predictive factors for the presence of amyloid and assess the degree of cardiac involvement in patients with amyloid deposition in the synovial tissue.

## 2. Materials and Methods

This prospective study, with longitudinal cohort follow-up, was carried out at the Parc Taulí University Hospital in Sabadell (Barcelona, Spain), a third-level teaching hospital.

The research was conducted by a multidisciplinary group of internal medicine specialists, radiologists, pathologists, cardiologists, and orthopedic surgeons specializing in hand surgery. All patients were informed before agreeing to participate in the study and provided signed informed consent before surgery.

Consecutive patients who underwent CTS surgery at our center were included. The inclusion period was from 1 January 2019, to 31 May 2021. All patients had a clinical and electromyographic diagnosis of CTS. Furthermore, all patients were non-responders to conservative treatment.

Patients underwent surgery using the mini-open technique on the axis of the fourth finger and the longitudinal section of the TCL at the most ulnar margin. After the release of the median nerve and flexor tendons, two samples were obtained (one from a segment of the ligament parallel to the opening on its radial side, and another from a segment of the synovial membrane on the superficial flexor tendon of the ring finger identified through active mobilization by the patient). The skin was closed with monofilament sutures and a semi-compressive bandage was applied in all cases.

**Figure 1:**
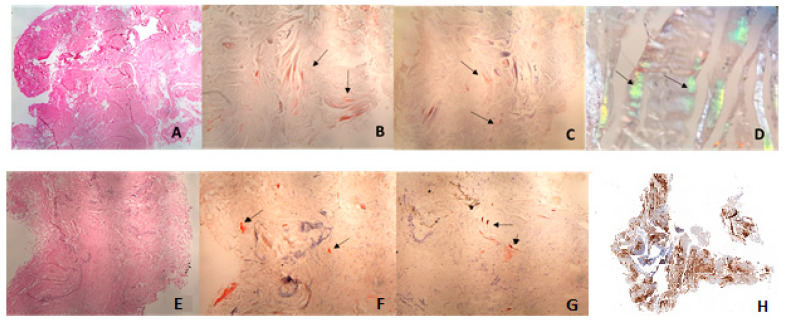
Biopsy of ligament (10×): hematoxylin–eosin (**A**) Congo-red positive (**B**) and Congo-red permanganate positive (**C**); apple green birefringence of amyloid deposit with polarized light (**D**) Biopsy of synovial membrane (10×): hematoxylin–eosin (**E**) Congo-red positive (**F**) and Congo-red permanganate positive (**G**), positive immunohistochemical study for anti-transthyretin antibodies (**H**). The meaning of the arrows is where it marks positivity for the stains described in the caption.

Samples from synovial tissue and CTL were studied. Synovial and CTL tissue samples were fixed in formalin, embedded in paraffin, and stained with hematoxylin and eosin. We employed a two-pronged approach, as follows: Congo red staining with permanganate pretreatment for a rapid and low-cost initial assessment, and immunohistochemistry for definitive confirmation A histochemical study was performed on each sample using Congo red with permanganate pretreatment and Congo red permanganate staining. An immunohistochemical study was also conducted with anti-amyloid A antibody (mc1, Diagnostic BioSystems, Pleasanton, CA, USA), anti-kappa chain (A21Y, Roche, Basel, Switzerland), anti-Lambda chain (K22Y, Roche), and anti-transthyretin (Polyclonal, Dako, Glostrup, Denmark) (Figure 1).

The previous medical history of patients was documented, as well as a physical exam performed before CTL surgery. The Charlson Comorbidity Index was used to account for the potential impact of comorbid conditions.

After obtaining the histopathological results, patients with evidence of amyloid deposition in the tenosynovial tissue and/or CTL underwent additional testing, including electrocardiography, blood and urine tests, echocardiography, and 99mTc-DPD scintigraphy, to assess the degree of cardiac involvement and to confirm the diagnosis of amyloidosis

Echocardiography was performed by a dedicated cardiologist with expertise in echocardiography using a Philips EPIQ 7C Digital Ultrasound System. Images were processed using the Intellispace Cardiovascular software version 4.2 (Philips Healthcare, Andover, MA, USA). The following parameters were analyzed: ventricular diameter, left ventricular wall thickness, left atrial volume, right atrial area, left and right ventricular function parameters, diastolic function, and transvalvular flow. Left ventricular longitudinal strain was also analyzed in 4-chamber, 2-chamber, and 3-chamber planes using AutoStrain LV software version 5.0 (Philips Healthcare). All of these items were assessed in accordance with the European Society of Cardiovascular Imaging and American Society of Echocardiography guidelines [22,23].

The nuclear medicine tests were performed using a Symbia Intevo double-head gamma camera (Siemens, Munich, Germany) with the following parameters: full-body images, LEHR collimator, 512 × 256 matrix, 3000 Kc; planar images of the thorax, LEHR collimator, 256 × 256 matrix, and 1000 Kc.99m. The 99mTc-DPD was infused intravenously, giving a total radiation dose of 925 megabecquerels. Full body images were obtained in anterior and posterior projections or selective images of the thorax in anterior projections, and in some cases in left anterior and left lateral oblique projections of 45° over the following two–three hours. A single radiologist and cardiologist interpreted the images. The diagnosis of myocardial uptake was defined according to the Perugini visual scale, which assesses the uptake of both ventricles compared to the adjacent rib. Grades 1 (less than rib uptake), 2 (same as rib uptake) and grade 3 (greater than rib uptake) were considered as a positive uptake [6].

The study was approved by the hospital’s ethics board (2019/677) and adhered to the Declaration of Helsinki principles. The original RCT was registered in ClinicalTrials.gov with registry identifier NCT04245098. The hospital and the ethics committee decided to assume the additional cost of the initial amyloid detection analysis (hematoxylin/eosin and Congo Red stain) due to its low cost.

### Statistical Analysis

Qualitative variables are expressed as percentages with their confidence intervals. Continuous variables are expressed as the mean ± standard deviation (SD). For the age variable, the median and 25th and 75th percentiles were used because their distribution was not normal. To determine the ability to discriminate between the diagnosis of amyloidosis and age, a receiver operating characteristic (ROC) curve was constructed, and the cut-off point for age with the greatest sensitivity and specificity was determined using the area under the curve and Youden index. Univariate comparisons between groups (patients with or without amyloid) were performed using the Chi-square test or Fisher’s exact test, depending on the nature of the variable investigated. Age was compared using the Mann–Whitney U test. Variables with *p* < 0.20 on univariate analysis were selected for inclusion in a multivariate logistic regression model. From the final logistic models, the odds ratios (ORs) and their 95% confidence intervals (CIs) for each variable are shown. Statistical significance was established at *p* < 0.05. The analyses were performed with the statistical program IBM^®^ SPSS^®^ version 28.

## 3. Results

### 3.1. Baseline Features and Presence of Amyloid

Two hundred forty-six patients were included in the study. CTL and synovial membrane samples were obtained from all the patients. The mean age was 60.2 years old, and 158 (64.23%) were women.

The most prevalent comorbidities in the patients were hypertension (130 patients, 52.85%), history of smoking (79, 32.11%), dyslipidemia (76, 30.89%), and diabetes mellitus (46, 18.70%).

In 134 patients (54.47%), a cause associated with carpal tunnel syndrome was detected, as follows: work history associated with CTS in 124 (50.41%), alcoholism in 14 (5.69%), hypothyroidism in 14 (5.69%), and connective tissue disease in 5 (2.03%).

Regarding the clinical factors associated with CTS, 151 patients (61.38%) underwent surgery on the right side, 60 (24.39%) had a history of contralateral surgery, and 27 (10.98%) had a trigger finger. No complications occurred following the surgical procedure.

Amyloid material was detected in CTL in 28 patients (11.38%) and in 31 (12.60%) synovial membrane samples. Amyloid was detected only in the synovial membranes of three patients.

Regarding immunohistochemistry, all patients were positive for transthyretin, except one, who was resistant to permanganate, positive for lambda light chains, and negative for kappa and amyloid A.

### 3.2. Characteristics of Patients with Amyloid in the Biopsy

Amyloid material was detected in 28 of the 88 patients aged >65 years (31.82%).

The baseline demographic characteristics, comorbidities, and pharmacological treatment of all 246 patients according to the presence (*n* = 31, 12.6%) or absence (*n* = 215, 87.4%) of amyloid in the synovial membrane biopsy are shown in Table 1. Patients with a positive biopsy were older (median age 58.04 years vs. 75.39, *p* < 0.001). The cut-off point identified by the Youden index (0.694) that simultaneously maximized the sensitivity (0.903) and specificity (0.721) was 64.50 years. Differentiating by sex, the cut-off point in men was 69.50 years (Youden 0.678, sensitivity 0.875 and specificity 0.803) and in women 64.50 years (Youden 0.618, sensitivity 0.933 and specificity 0.685). Although the presence of amyloid was more frequent in males, there were no significant differences in our cohort in terms of sex.

**Figure 2:**
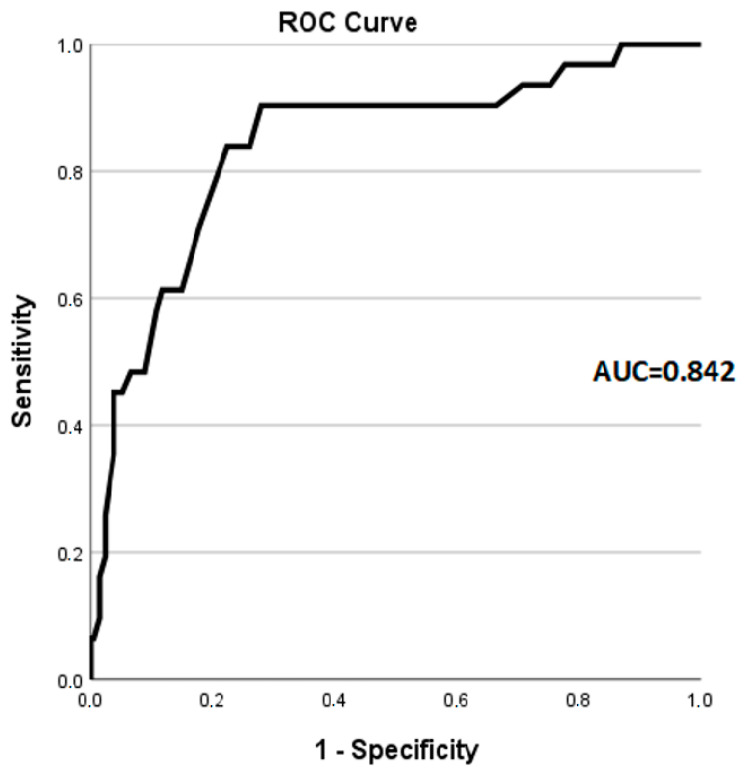
Receiver operating characteristic (ROC) curve for age as a predictor of amyloid positivity in carpal tunnel syndrome patients undergoing synovial membrane biopsy. (AUC = Area under curve).

The receiver operating characteristic (ROC) curve for age as a predictor of amyloid positivity in carpal tunnel syndrome patients undergoing synovial membrane biopsy is shown in Figure 2.

The group with the presence of amyloid presented higher rates of heart failure (19.6% vs. 0%, *p* = 0.001), hypertensive heart disease (9.7% vs. 0.5%, *p* < 0.001), narrow lumbar canal (12.9% vs. 2.8%, *p* < 0.008), and atrial fibrillation (9.7% vs. 2.3%, *p* = 0.031). No differences were observed in the rest of the comorbidities.

The clinical characteristics of CTS, the possible etiologies and the size of the biopsies according to the presence or absence of amyloid are shown in Table 2. No differences were observed in terms of whether the right or left side was operated on, whether the intervention was on the dominant or non-dominant hand, or the time between the onset of symptoms and surgery.

The following possible etiologies of CTS were evaluated: thyroid pathology, DM, acromegaly, work history associated with CTS, connective tissue diseases, acute trauma, alcoholism, diagnosis of b12 deficiency, pregnancy, and treatment with anovulatory and neurotoxic drugs (isoniazid, antabuse, amiodarone, metronidazole, and cytostatics). No statistically significant differences were found between the groups.

Patients with amyloid in the biopsy presented with more bilateral CTS symptoms (87.1% vs. 65.6%, *p* = 0.016), more previous contralateral carpal surgery (41.9% vs. 29.9%, *p* = 0.015), and more trigger finger (29.9% vs. 8.4%) *p* < 0.001). Regarding the size of the extracted biopsy, no differences were observed in either CTL or synovial membrane.

Patients with amyloid on biopsy underwent additional laboratory tests, echocardiography, and cardiac scintigraphy. The characteristics of these patients, differentiated according to the presence or absence of myocardial uptake on scintigraphy, are presented in Table 3.

Of these 31 patients, 13 (41.94%) presented with some degree of myocardial uptake and 18 (58.06%) did not. Of the 13 patients with myocardial uptake, 5 (38.46%) presented grade I, 3 grade II and 5 (38.46%) grade III.

The group with cardiac uptake were older (80.46 ± 6.85 vs. 71.72 ± 12.16 years) and presented higher rates of heart failure (46.15% vs. 0%), arterial hypertension (76.92% vs. 33.33%), and atrial fibrillation (23.08% vs. 0%). N-terminal ProBNP levels were higher in patients with cardiac uptake (675.62 ± 998.86 pg/mL vs. 159.30 ± 143.61 pg/mL). Echocardiographic findings showed that the cardiac uptake group had a higher prevalence of increased left ventricular wall thickness (46.15% vs. 17.67%) and low ejection fraction (15.38% vs. 5.56%). There were also more cases of diastolic alteration in the cardiac uptake group (69.23% vs. 11.11%). The prevalence of aortic stenosis was slightly higher in the cardiac uptake group (7.69% vs. 5.56%). No differences were observed in other comorbidities such as diabetes mellitus, spinal lumbar stenosis, and trigger finger.

After completing the supplementary investigations, 17 patients were diagnosed with amyloidosis due to transthyretin located in the carpus (no scintigraphic cardiac uptake or grade 0); 1 with immunoglobulin light chain amyloidosis; 8 with ATTR with cardiac involvement (grade 2–3); and 5 with transthyretin amyloidosis in the carpus presenting grade 1 scintigraphic uptake, without abnormalities on laboratory tests or echocardiogram. None of the patients had a transthyretin-associated mutation.

The individual characteristics of patients with myocardial uptake and echocardiogram values are shown in Table 4.

### 3.3. Predictors of the Presence of Amyloid in the Biopsy

All demographic factors, comorbidities, etiological factors, and clinical factors presented in Table 1 and Table 2 were assessed as potential predictors of the presence of amyloid in the biopsy. Factors with *p* values below 0.20 were age, male sex, heart failure, atrial fibrillation, valvular heart disease, hypertensive heart disease, treatment with diuretics, digoxin, anticoagulants, a narrow lumbar canal, bilateral CTS symptoms, previous surgery of the contralateral carpus, and trigger finger.

In the multivariate analysis, the following independent predictors of amyloid were detected: age (*p* = 0.035 OR 1.123, 1.076–1.172), bilateral CTS symptoms (*p* = 0.022 OR 3.647, 1.093–12.168), and trigger finger (*p* < 0.001 OR 3.537, 1.204–10.392) (Table 5).

## 4. Discussion

We report a large sample of patients undergoing carpal tunnel surgery, in whom biopsies of the CTL and synovial membrane were performed for the detection of amyloid. Amyloid was detected in 12.6% of biopsies. Most patients had ATTR without cardiac involvement. Age, bilateral symptoms, and trigger finger were independent predictors of a positive biopsy result.

Previous studies analyzing the presence of amyloid material during CTS surgery have reported variable rates, ranging from 2.3% to 56%. The main reason for this difference is the heterogeneity of the cohorts, mainly due to the age, race, and sex of the included patients [24,25,26,27,28,29,30,31,32].

The prevalence of amyloidosis increases with age, but previous research has not established a cut-off point to indicate the age at which CTS biopsies should be performed to detect the presence of amyloid. In our study, we identified age as a significant predictor of amyloid in carpal tunnel syndrome patients undergoing synovial membrane biopsy. Employing the Youden index, we identified an age cut-off of 64.5 years for amyloidosis detection. At this cut-off, sensitivity and specificity were 90.3% and 72.1%, respectively. The area under the ROC curve (AUC) was 0.843, indicating moderate discriminatory power. These data suggest that assessing biopsy according to age (alone or in combination with other factors) could be useful to optimize the diagnostic yield for amyloid in patients in patients undergoing carpal tunnel surgery.

There are no guidelines regarding the type of tissue that should be biopsied for amyloidosis diagnosis. Some authors have suggested a higher prevalence of amyloid in the carpal tunnel ligament than in the synovial membrane [28]. In our study, both tissues were biopsied from each patient, and a slightly higher frequency of amyloid deposits was found in the synovial membrane. Therefore, obtaining samples from both tissues in a standardized manner may be beneficial for a more comprehensive diagnosis, particularly when considering the potential variability in amyloid deposition.

Additionally, immunohistochemical staining revealed that the vast majority of biopsies were positive for transthyretin. This finding highlights the predominance of ATTR amyloidosis in our patient population.

A history of bilateral symptoms or bilateral carpal tunnel release surgery was considered as a red flag. Some studies suggest that a history of previous surgery on the contralateral carpus is more frequent in patients with the presence of amyloid [24]. Our data suggest the presence of clinical symptoms that demonstrate this association. Therefore, bilateral clinical CTS should be considered an alarm sign in this population.

Flexor-stenosing tenosynovitis (also known as trigger finger) is also common in patients with amyloidosis. The prevalence of amyloid in patients with trigger finger is variable, ranging between 2% and 65% [33,34,35,36]. According to our data, when trigger finger occurs concomitantly with CTS, it is a predictor of the presence of amyloid that clinicians should consider.

Our findings are relevant because, in this prospective study with a relatively large cohort, we were able to show the prevalence of amyloid in CTS of 12.6% of patients undergoing CTS surgery. If not performed as a standardized protocol, additional pathology examinations and clinical investigations could be considered in patients with CTS presenting any of the risk factors for the presence of amyloid (age > 64.5 years and/or bilateral symptoms and/or trigger finger). This could lead to an earlier diagnosis of amyloidosis and the earlier introduction of effective therapies that delay the development of end-stage cardiac disease.

### Study Limitations

Among the limitations of our study, our patients were only Caucasian, single proton emission computerized tomography/computerized tomography imaging was not included, and there was no long-term follow-up. Otherwise, this is the largest series of patients with a systematic prospective recruitment protocol that also identifies predictors of amyloid presence.

## 5. Conclusions

We detected amyloid in 12.6% of unselected consecutive patients who underwent CTS surgery. Biopsy in patients with CTS for amyloid detection, especially in elderly patients with bilateral symptoms and trigger finger, may be useful for the early diagnosis of amyloidosis, primarily due to transthyretin.

## Figures and Tables

**Table 1:** Baseline characteristics, comorbidities and pharmacological history.

	No Amyloid (*n* = 215)	Amyloid (*n* = 31)	*p* Value
Demographic data			
Age (years ± SD)	58.04 ± 12.64 E 0.862	75.39 ± 11.041 E 1.983	*p* < 0.001 *
Gender: female/male	142 (66%)/73 (34%)	16 (51.6%)/15 (48.4%)	0.117 *
Weight (kg ± SD)	75.93 ± 13.77	73.171 ± 10.63 E 1.909	0.285
Height (M)	1.64 ± 0.089	1.63 ± 0.0084	0.624
Body mass index	28.13 ± 4.63 E 0.315	27.49 ± 3.85 E 0.69	0.437
Comorbidities			
Smoking history	72 (33.49%)	7 (22.58%)	0.224
Diabetes mellitus	41 (19.5%)	5 (16.1%)	0.695
Arterial hypertension	111 (51.6%)	19 (61.3%)	0.314
Chronic obstructive pulmonary disease	18 (8.4%)	2 (6.5%)	0.715
Heart failure	0 (0%)	6 (19.4%)	<0.001 *
Valvular heart disease	3 (1.4%)	2 (6.5%)	0.062 *
Ischemic heart disease	12 (5.6%)	1 (3.2%)	0.584
Hypertensive heart disease	1 (0.5%)	3 (9.7%)	<0.001 *
Stroke	8 (3.7%)	1 (3.2%)	0.891
Peripheral vasculopathy	24 (11.2%)	5 (16.1%)	0.423
Dyslipidemia	65 (30.2%)	11 (35.4%)	0.554
Non-metastatic neoplasm	6 (2.8%)	2 (6.5%)	0.283
Metastatic neoplasm	7 (3.3%)	0 (0%)	0.308
Renal insufficiency	7 (3.3%)	1 (3.2%)	0.993
Spinal lumbar stenosis	6 (2.8%)	4 (12.9%)	0.008 *
Atrial fibrillation	5 (2.3%)	3 (9.7%)	0.031 *
Charlson	0.93	0.87	0.841
Drugs			
Angiotensin-converting enzyme inhibitors	83 (38.6%)	14 (45.4%)	0.485
Angiotensin II receptor antagonists	27 (12.6%)	5 (16.1%)	0.581
Calcium antagonists	19 (8.8%)	3 (9.7%)	0.878
Thiazides	27 (12.6%)	7 (22.6%)	0.131
Beta blockers	10 (4.7%)	2 (6.5%)	0.664
Acetylsalicylic acid	9 (4.2%)	2 (6.5%)	0.568
Digoxin	1 (0.5%)	1 (3.2%)	0.11 *
Anticoagulants	8 (3.7%)	4 (12.9%)	0.026 *
Furosemide	8 (3.7%)	5 (16.1%)	0.004 *

* Variables included in the multivariate analysis.

**Table 2:** Clinical factors, comorbidities and biopsy associated with carpal tunnel syndrome.

	No Amyloid (*n* = 215)	Amyloid (*n* = 31)	*p* Value
Causes of carpal tunnel syndrome			
Alcoholism	14 (6.5%)	0 (0%)	0.143
Hypothyroidism	13 (6%)	1 (3.2%)	0.526
Associated work history	107 (49.8%)	17 (54.8%)	0.598
Connective tissue disease	5 (2.3%)	0 (0%)	0.391
Acute trauma	2 (0.9%)	0 (0%)	0.59
Clinical factors associated with CTS			
Side: right/left	131 (60.9%)/84 (39.1%)	20 (64.5%)/11 (35.5%)	0.701
Dominant hand intervened	173 (80.5%)	24 (77.4%)	0.691
Bilateral clinical symptoms	141 (65.6%)	27 (87.1%)	0.016 *
History of contralateral surgery	47 (29.9%)	13 (41.9%)	0.015 *
Trigger finger	18 (8.4%)	9 (29.9%)	*p* < 0.001 *

* Variables included in the multivariate analysis.

**Table 3:** Characteristics of patients with the presence of amyloid in the biopsy classified according to the presence or absence of cardiac uptake in scintigraphy.

	No Uptake (*n* = 18)	Uptake (*n* = 13)
Demographic data		
Age (years ± SD)	71.72 ± 12.16	80.46 ± 6.85
Gender: female/male	10 (55.56%)/8 (44.44%)	6 (46.15%)/7 (53.85%)
Comorbidities		
Diabetes mellitus	3 (16.67%)	2 (15.38%)
Arterial hypertension	9 (50%)	10 (76.92%)
Heart failure	0 (0%)	6 (46.15%)
Spinal lumbar stenosis	3 (16.67%)	1 (7.69%)
Atrial fibrillation	0 (0%)	3 (23.08%)
Charlson	0.56 ± 0.71	1.31 ± 1.18
Clinical factors associated with STC		
Side: right/left	11 (61.11%)/7 (38.89%)	9 (69.23%)/4 (30.77%)
Bilateral clinical symptoms	17 (94%)	10 (76.92%)
History of contralateral surgery	7 (38.89%)	6 (46.15%)
Trigger finger	6 (33.33%)	3 (23.08%)
Time of onset until surgery (days)	1782.44 ± 1606.11	1939.77 ± 1276.23
Electrocardiogram		
Atrial fibrillation	0 (0%)	3 (23.08%)
Right bundle branch block	0 (0%)	1 (7.69%)
Left bundle branch block	0 (0%)	1 (7.69%)
Analytical values		
Hemoglobin (g/L)	138.83 ± 13.738	136 ± 16.907
Creatinine (mg/dL)	0.83 ± 0.216	1.00 ± 0.39
Sodium (mEq/L)	140.33 ± 2.19	141 ± 1.96
Potassium (mEq/L)	4.55 ± 0.34	4.3 ± 0.35
N-terminal ProBNP (pg/mL)	159.50 ± 143.61	695.62 ± 998.96
Troponin T u.s (ng/L)	14.15 ± 9.19	23.85 ± 17.95
Monoclonal immunoglobulin	1 (5.56%)	0 (0%)
Positive genetic test	0 (0%)	0 (0%)
Echocardiogram		
Left ventricular wall thickness ≥ 12 mm	3 (17.67%)	6 (46.15%)
Low ejection fraction (<50)	1 (5.56%)	2 (15.38%)
Diastolic alteration	2 (11.11%)	9 (69.23%)
Aortic stenosis	1 (5.56%)	1 (7.69%)
Grade of myocardiac uptake on scintigraphy		
Grade 0	18 (100%)	0 (0%)
Grade 1	0 (0%)	5 (38.46%)
Grade 2	0 (0%)	3 (23.08%)
Grade 4	0 (0%)	5 (38.46%)

**Table 4:** Individual characteristics of the patients with myocardial uptake and the echocardiogram parameters.

Patient	1	2	3	4	5	6	7	8	9	10	11	12	13
Age	83	84	81	82	92	66	77	83	86	82	85	72	73
Gender	Female	Male	Female	Female	Male	Male	Male	Female	Male	Female	Male	Female	Male
Cardiac biomarkers													
Pro Bnp	235	157	448	444	2846	105	286	200	2788	67	1263	69	206
Troponin	20	11	24	20	56	12	34	11	62	10	32	15	17
EKG													
Rhythm	Sinus	Sinus	Sinus	Sinus	AF	Sinus	Sinus	sinus	Sinus	Sinus	Sinus	AF	Sinus
Altered conduction	No	No	No	No	RBBB	No	No	No	LBBB	No	No	No	No
ECHO parameters													
VST (mm)	1.22	1.03	1.07	1.03	1.62	1.15	1.25	1.01	1.47	1.15	1.48	1.09	1.33
LVPW (mm)	1.20	0.92	0.98	0.96	1.48	0.88	0.93	0.93	1	0.89	1.34	1.16	1.27
LVEF (%)	60	60.4	60	60	55	65	60	60	32	63	65	65	63
Diastolic stage	1	Normal	2	2	NA	Normal	2	2	3	NA	2	NA	Normal
LAV (L/m^2^)	38.6	27.9	48.5	49.5	43.7	30.2	45.7	42.2	51.2	27.2	34.5	37.4	18.8
E/A VM	0.77	0.60	0.71	0.70	0.73	0.82	0.70	1.26	2.5	0.56	0.81	0.73	0.61
Average E/e’	12.65	6.6	13.2	13.4	17.75	6.35	14.6	14.15	20.7	13.25	14.05	10.9	8.05
Overall strain value	−18	−19.4	−21.7	−21.9	−12.5	−21	−21.9	−23	−5	NA	−18.8	−15	−21
Valvular involvement	No	No	No	No	No	Aortic stenosis	No	Aortic stenosis	Aortic stenosis	No	Aortic stenosis	No	No
Scintigraphy (Grade of uptake)	3	1	1	1	3	3	3	2	2	1	3	2	1

EKG: electrocardiogram, ECHO: echocardiogram, VST: ventricular septal thickness, LVPW: left ventricular posterior wall, LVEF: left ventricular ejection fraction, LAV: left atrial volume, E/A VM: transmitral flow ratio, NA: not assessed AF: atrial fibrillation LBBB: left bundle branch block RBBB: right bundle branch block.

**Table 5:** Predictive factors of positive biopsy for amyloid.

	*p* Value	Odds Ratio
Age	0.035	1.123 (1.076–1.172)
Bilateral symptoms	0.022	3.647 (1.093–12.168)
Trigger finger	*p* < 0.001	3.537 (1.204–10.392)

## Data Availability

The authors confirm that the data supporting the findings of this study are available within the article.

## References

[1] García-Pavía P., Tomé-Esteban M.T., Rapezzi C. (2011). Amiloidosis. También una enfermedad del corazón [Amyloidosis. Also a heart disease]. Rev. Esp. Cardiol..

[2] Rapezzi C., Lorenzini M., Longhi S., Milandri A., Gagliardi C., Bartolomei I., Salvi F., Maurer M.S. (2015). Cardiac amyloidosis: The great pretender. Heart Fail. Rev..

[3] Vranian M.N., Sperry B.W., Valent J., Hanna M. (2015). Emerging Advances in the Management of Cardiac Amyloidosis. Curr. Cardiol. Rep..

[4] Maurer M.S., Elliott P., Comenzo R., Semigran M., Rapezzi C. (2017). Addressing Common Questions Encountered in the Diagnosis and Management of Cardiac Amyloidosis. Circulation.

[5] De Gregorio C., Trimarchi G., Faro D.C., De Gaetano F., Campisi M., Losi V., Zito C., Tamburino C., Di Bella G., Monte I.P. (2023). Myocardial Work Appraisal in Transthyretin Cardiac Amyloidosis and Nonobstructive Hypertrophic Cardiomyopathy. Am. J. Cardiol..

[6] Aimo A., Fabiani I., Giannoni A., Mandoli G.E., Pastore M.C., Vergaro G., Spini V., Chubuchny V., Pasanisi E.M., Petersen C. (2022). Multi-chamber speckle tracking imaging and diagnostic value of left atrial strain in cardiac amyloidosis. Eur. Heart J. Cardiovasc. Imaging.

[7] Gillmore J.D., Maurer M.S., Falk R.H., Merlini G., Damy T., Dispenzieri A., Wechalekar A.D., Berk J.L., Quarta C.C., Grogan M. (2016). Nonbiopsy Diagnosis of Cardiac Transthyretin Amyloidosis. Circulation.

[8] Perugini E., Guidalotti P.L., Salvi F., Cooke R.M., Pettinato C., Riva L., Leone O., Farsad M., Ciliberti P., Bacchi-Reggiani L. (2005). Noninvasive etiologic diagnosis of cardiac amyloidosis using 99mTc-3,3-diphosphono-1,2-propanodicarboxylic acid scintigraphy. J. Am. Coll. Cardiol..

[9] Sant’Anna R., Gallego P., Robinson L.Z., Pereira-Henriques A., Ferreira N., Pinheiro F., Esperante S., Pallares I., Huertas O., Almeida M.R. (2016). Repositioning tolcapone as a potent inhibitor of transthyretin amyloidogenesis and associated cellular toxicity. Nat. Commun..

[10] Coelho T., Adams D., Silva A., Lozeron P., Hawkins P.N., Mant T., Perez J., Chiesa J., Warrington S., Tranter E. (2013). Safety and efficacy of RNAi therapy for transthyretin amyloidosis. N. Engl. J. Med..

[11] Suhr O.B., Coelho T., Buades J., Pouget J., Conceicao I., Berk J., Schmidt H., Waddington-Cruz M., Campistol J.M., Bettencourt B.R. (2015). Efficacy and safety of patisiran for familial amyloidotic polyneuropathy: A phase II multi-dose study. Orphanet J. Rare Dis..

[12] Cornwell G.G., Murdoch W.L., Kyle R.A., Westermark P., Pitkänen P. (1983). Frequency and distribution of senile cardiovascular amyloid. A clinicopathologic correlation. Am. J. Med..

[13] Tanskanen M., Peuralinna T., Polvikoski T., Notkola I.L., Sulkava R., Hardy J., Singleton A., Kiuru-Enari S., Paetau A., Tienari P.J. (2008). Senile systemic amyloidosis affects 25% of the very aged and associates with genetic variation in alpha2-macroglobulin and tau: A population-based autopsy study. Ann. Med..

[14] Milandri A., Farioli A., Gagliardi C., Longhi S., Salvi F., Curti S., Foffi S., Caponetti A.G., Lorenzini M., Ferlini A. (2020). Carpal tunnel syndrome in cardiac amyloidosis: Implications for early diagnosis and prognostic role across the spectrum of aetiologies. Eur. J. Heart Fail..

[15] Zhang D., Makhni M.C., Kang J.D., Blazar P. (2021). Orthopaedic Manifestations of Amyloidosis. J. Am. Acad. Orthop. Surg..

[16] Atroshi I., Gummesson C., Johnsson R., Ornstein E., Ranstam J., Rosén I. (1999). Prevalence of carpal tunnel syndrome in a general population. AMA.

[17] González-López E., López-Sainz Á., Garcia-Pavia P. (2017). Diagnosis and Treatment of Transthyretin Cardiac Amyloidosis. Progress and Hope. Rev. Esp. Cardiol. (Engl. Ed.).

[18] Rapezzi C., Merlini G., Quarta C.C., Riva L., Longhi S., Leone O., Salvi F., Ciliberti P., Pastorelli F., Biagini E. (2009). Systemic cardiac amyloidoses: Disease profiles and clinical courses of the 3 main types. Circulation.

[19] Prokaeva T., Spencer B., Kaut M., Ozonoff A., Doros G., Connors L.H., Skinner M., Seldin D.C. (2007). Soft tissue, joint, and bone manifestations of AL amyloidosis: Clinical presentation, molecular features, and survival. Arthritis Rheum..

[20] Kyle R.A., Greipp P.R. (1983). Amyloidosis (AL). Clinical and laboratory features in 229 cases. Mayo Clin. Proc..

[21] Donnelly J.P., Hanna M., Sperry B.W., Seitz W.H. (2019). Carpal Tunnel Syndrome: A Potential Early, Red-Flag Sign of Amyloidosis. J. Hand Surg..

[22] Lang R.M., Badano L.P., Mor-Avi V., Afilalo J., Armstrong A., Ernande L., Flachskampf F.A., Foster E., Goldstein S.A., Kuznetsova T. (2015). Recommendations for cardiac chamber quantification by echocardiography in adults: An update from the American Society of Echocardiography and the European Association of Cardiovascular Imaging. J. Am. Soc. Echocardiogr..

[23] Nagueh S.F., Smiseth O.A., Appleton C.P., Byrd B.F., Dokainish H., Edvardsen T., Flachskampf F.A., Gillebert T.C., Klein A.L., Lancellotti P. (2016). Recommendations for the Evaluation of Left Ventricular Diastolic Function by Echocardiography: An Update from the American Society of Echocardiography and the European Association of Cardiovascular Imaging. Eur. Heart J. Cardiovasc. Imaging.

[24] Grogan M., Scott C.G., Kyle R.A., Zeldenrust S.R., Gertz M.A., Lin G., Klarich K.W., Miller W.L., Maleszewski J.J., Dispenzieri A. (2016). Natural History of Wild-Type Transthyretin Cardiac Amyloidosis and Risk Stratification Using a Novel Staging System. J. Am. Coll. Cardiol..

[25] Damy T., Costes B., Hagège A.A., Donal E., Eicher J.C., Slama M., Guellich A., Rappeneau S., Gueffet J.P., Logeart D. (2016). Prevalence and clinical phenotype of hereditary transthyretin amyloid cardiomyopathy in patients with increased left ventricular wall thickness. Eur. Heart J..

[26] Sperry B.W., Reyes B.A., Ikram A., Donnelly J.P., Phelan D., Jaber W.A., Shapiro D., Evans P.J., Maschke S., Kilpatrick S.E. (2018). Tenosynovial and Cardiac Amyloidosis in Patients Undergoing Carpal Tunnel Release. J. Am. Coll. Cardiol..

[27] Zegri-Reiriz I., de Haro-Del Moral F.J., Dominguez F., Salas C., de la Cuadra P., Plaza A., Krsnik I., Gonzalez-Lopez E., Garcia-Pavia P. (2019). Prevalence of Cardiac Amyloidosis in Patients with Carpal Tunnel Syndrome. J. Cardiovasc. Transl. Res..

[28] Hahn K., Urban P., Meliß R.R., Axmann H.D., Siebert F., Röcken C. (2018). Karpaltunnelsyndrom und ATTR-Amyloidose [Carpal tunnel syndrome and ATTR-amyloidosis]. Handchir. Mikrochir. Plast. Chir..

[29] Sekijima Y., Uchiyama S., Tojo K., Sano K., Shimizu Y., Imaeda T., Hoshii Y., Kato H., Ikeda S. (2011). High prevalence of wild-type transthyretin deposition in patients with idiopathic carpal tunnel syndrome: A common cause of carpal tunnel syndrome in the elderly. Hum. Pathol..

[30] Vianello P.F., La Malfa G., Tini G., Mazzola V., Miceli A., Santolini E., Briano S., Porto I., Canepa M. (2020). Prevalence of transthyretin amyloid cardiomyopathy in male patients who underwent bilateral carpal tunnel surgery: The ACTUAL study. Int. J. Cardiol..

[31] Scott K.L., Conley C.R., Renfree K.J. (2019). Histopathologic Evaluation of Flexor Tenosynovium in Recurrent Carpal Tunnel Syndrome. Plast. Reconstr. Surg..

[32] Westin O., Fosbøl E.L., Maurer M.S., Leicht B.P., Hasbak P., Mylin A.K., Rørvig S., Lindkær T.H., Johannesen H.H., Gustafsson F. (2022). Screening for Cardiac Amyloidosis 5 to 15 Years after Surgery for Bilateral Carpal Tunnel Syndrome. J. Am. Coll. Cardiol..

[33] Cordiner-Lawrie S., Diaz J., Burge P., Athanasou N.A. (2001). Localized amyloid deposition in trigger finger. J. Hand Surg..

[34] Uotani K., Kawata A., Nagao M., Mizutani T., Hayashi H. (2007). Trigger finger as an initial manifestation of familial amyloid polyneuropathy in a patient with Ile107Val TTR. Intern. Med..

[35] Hara Y., Tajiri Y., Kawano K., Hoshikawa S., Kita Y. (2020). The Tenosynovitis of Fingers Associated with Transthyretin Amyloidosis. J. Hand Surg. Asian Pac. Vol..

[36] Sperry B.W., Khedraki R., Gabrovsek A., Donnelly J.P., Kilpatrick S., Shapiro D., Evans P.J., Maschke S., Cotta C., Nakashima M. (2021). Cardiac Amyloidosis Screening at Trigger Finger Release Surgery. Am. J. Cardiol..

